# Study on Drying Characteristics of Juvenile Wood of *Dalbergia odorifera* T.C.Chen

**DOI:** 10.3390/ma19112234

**Published:** 2026-05-25

**Authors:** Jia Liu, Tongtong Li, Jianing Li, Honghai Liu

**Affiliations:** 1 Jiangsu Co-Innovation Center of Efficient Processing and Utilization of Forest Resources, Nanjing Forestry University, Nanjing 210037, China; jia@njfu.edu.cn; 2 Rubber Research Institute, Chinese Academy of Tropical Agricultural Sciences, Haikou 571101, China; tongxinltt@163.com; 3 College of Furnishings and Industrial Design, Nanjing Forestry University, Nanjing 210037, China

**Keywords:** *Dalbergia odorifera*, drying characteristics, drying schedule optimization, drying quality

## Abstract

*Dalbergia odorifera* is an economically valuable timber species. While its plantation-grown stock displays unique juvenile wood characteristics, current drying research is insufficient and lacks direct applicability to the heartwood. This study investigated the drying characteristics of a 17-year-old plantation-grown *Dalbergia odorifera* from Hainan using the 100 °C drying test method. Conventional drying technology was optimized by adjusting key parameters, including drying rate, moisture content variation, residual stress, and drying defects. The results showed that the overall drying defect grade of 25 mm-thick *Dalbergia odorifera* specimens were Grade 3, and the superior drying quality was achieved under relatively low-temperature and high-humidity conditions. The optimal drying schedule was comprehensively determined as follows: an initial temperature of 48 °C with a wet-bulb depression of 3 °C, a final temperature of 68 °C with a wet-bulb depression of 12 °C, and a drying duration of approximately 12 days to reduce the moisture content (MC) from 50% to 10%. Under this schedule, all drying quality indicators of the sawn timber met Grade 2 standards, and the color of wood has been well preserved. These results provided a theoretical and technical reference for improving the utilization efficiency of wood and the industrial drying of plantation-grown *Dalbergia odorifera*.

## 1. Introduction

*Dalbergia odorifera* T.C.Chen, namely Scented rosewood or Hainan Huanghuali, is an endemic tree species to Hainan, China, belonging to the Dalbergia genus of the Fabaceae family. Its heartwood is recognized as one of the precious rosewoods in China [1]. Distinguished by high hardness, density and abundant extractives, the *Dalbergia odorifera* exhibits an elegant wood grain. The fresh cross-sections of its heartwood present a red to reddish-brown color and emit a distinct spicy aroma [2], exhibiting excellent visual and olfactory properties. These characteristics contribute to its significant application value in the production of premium furniture and artisanal crafts [3]. Moreover, both the wood and its extractives are suitable for application in the extraction of spices [4] and the sources of medicinal components [5,6], further enhancing the economic value.

The heartwood formation period of *Dalbergia odorifera* is relatively long, usually taking about 7 years to start forming heartwood, and the maturation time is even more 30 to 40 years [7]. Due to the continuous consumption of wild resources and the control of international trade regulations and other factors [8], the large-diameter natural *Dalbergia odorifera* has become extremely scarce in the market, where the timber currently utilized is predominantly young-grown and small-diameter wood from artificial plantations. Since the 1960s, *Dalbergia odorifera* has been successfully introduced for artificial cultivation in Guangdong, Guangxi, Sichuan, Guizhou, Fujian and other regions with a climate similar to that of Hainan [9]. In recent years, with the expanding plantation scale of *Dalbergia odorifera* in Hainan, the artificial plantation-grown timber has gradually become an important source of this wood.

For naturally grown rosewoods such as *Dalbergia odorifera*, their wood properties are relatively stable, and the good drying quality can typically be achieved by the conventional drying protocols of rosewood [10]. However, the plantation-grown *Dalbergia odorifera* exhibits significant differences from its natural counterpart in terms of growth age, wood properties, and wood color. The traditional drying methods are difficult to balance the drying efficiency and quality control effectively, presenting notable technical challenges. Consequently, systematic research on its drying techniques and optimal process parameters for plantation-grown material is urgently needed [10]. Most of the existing studies on plantation-grown *Dalbergia odorifera* have focused on the formation mechanisms of heartwood [11] and the chemical composition characteristics [12], while the research regarding its drying properties and processes remains relatively scarce. In particular, there is a lack of detailed analysis of drying behavior patterns based on clear tree age and background conditions of artificial plantation.

*Dalbergia odorifera* exhibits higher density and more abundant tyloses compared to the other common hardwoods [13], and the content of secondary metabolites such as flavonoid and terpenoid secondary metabolites in the heartwood is higher [14]. During the drying process, moisture migration is primarily governed by the connectivity of the vessel-pit system. Obstructions in the microscopic wood pathways reduce the drying rate and tend to cause severe drying stress and shrinkage defects [15]. The common drying methods employed for rosewoods including *Dalbergia odorifera* are air drying, conventional kiln drying and vacuum drying. During the Ming and Qing dynasties, it was recorded that the air drying of rosewood could take 1 to 3 years [16], however the drying quality in this method is highly susceptible to the ambient conditions. *Pterocarpus soyauxii*, a species with properties similar to *Dalbergia odorifera*, exhibits excellent drying characteristics under air drying [17], but its slow drying rate limits the high-efficiency utilization of its heartwood. Current researches on the conventional drying of *Dalbergia odorifera* are limited to sapwood. Wu et al. [18] developed a conventional drying schedule for the sapwood of *Dalbergia odorifera* using the 100 °C test method. Due to the significant differences in density, tyloses and permeability between sapwood and heartwood, this schedule is not directly applied to the drying of heartwood. Furthermore, the differences in drying characteristics between heartwood and sapwood vary among rosewood species. For instance, Li et al. [19] found that the heartwood and sapwood of African rosewood could achieve similar drying quality under the same drying conditions. Because of the interspecific difference in wood cell wall structure and extractive contents, this finding cannot be directly applied to *Dalbergia odorifera*. This further highlights the necessity of conducting specialized research on the drying characteristics of the heartwood of plantation-grown *Dalbergia odorifera*.

In this study, a 17-year-old plantation-grown *Dalbergia odorifera* from Hainan was selected to systematically investigate its drying characteristics by using the 100 °C experimental method. Based on the findings, the drying parameters were optimized to propose a drying protocol suitable for the plantation-grown *Dalbergia odorifera* by comparing the drying rates, moisture content changes, residual stress indicators and the drying defects during among each drying stage. This study would provide a theoretical basis and technical support for the drying technology of plantation-grown *Dalbergia odorifera* and ensure the high-value utilization of plantation-grown *Dalbergia odorifera*.

## 2. Materials and Methods

### 2.1. Materials

The plantation-grown *Dalbergia odorifera* specimens used in this study were provided by the rubber wood and tropical forest farm of Chinese Academy of Tropical Agricultural Sciences in Danzhou, Hainan Province, China. The trees were approximately 17 years old, with a breast-height diameter of 16 cm, a total height of 4–5 m and a branch height of 2.1 m. The trees had been transplanted twice. The initial moisture content (MC) of the heartwood was about 75%, and the average air-dried density (at 12% MC) was 0.74 g/cm^3^.

### 2.2. Equipment and Devices

The primary experimental equipment used in this study included a constant temperature and humidity chamber (BPS-250CA, Shang-hai Yiheng Scientific Instrument Co., Ltd., Shanghai, China), an electric oven (DHG-9070, Shang-hai Yiheng Scientific Instrument Co., Ltd., Shanghai, China), an electronic balance (ME204E, Mettler-Toledo, Greifensee, Switzerland), a scanner (CanoScan LiDE 120, CanonChina Co., Ltd., Beijing, China), a colorimeter (YG60s, Guangdong Snh Technology Co., Ltd., Guangzhou, China), a calibrated vernier caliper (ARZ-1331, Mitutoyo, Kawasaki, Japan).

### 2.3. Methods

#### 2.3.1. 100 °C Experimental Method

Nine heartwood specimens (heartwood ratio ≥ 90%) without obvious defects such as discoloration, cracks, knots and insect holes and other obvious defects were cut from the straight stem sections of each log, which were used as the test materials for the 100 °C drying experimental method. The specimens dimensions were sized at 100 mm (T) × 20 mm (R) × 200 mm (L). Six tangential boards were assigned to the experimental group and two radial boards and one central board were served as the control group [20].

The sawn specimens were dried at 103 ± 2 °C. During the drying process, the weight of specimens (m_i_) and the drying cracks (including end cracks, end-surface cracks, surface cracks, and through cracks) were recorded at regular intervals. Drying was terminated when the weight difference between two consecutive weight measurements was less than 0.5%. After removing the specimens and allowing them to cool, the oven-dry weight (m) and twist value of specimens were measured. The twist value is defined as the height of the deviation of one corner from the plane when three corners of the specimen are in contact with the plane. After measurement, a 15 mm-wide MC specimen was cut from the middle of the test piece along its length direction. The MC of the specimen was measured by the oven-drying method according to GB/T 1927.4-2021 [21], which was taken as the final MC (W_0_) of the entire test piece. The oven-dried weight (m_0_) of the test piece was calculated using Equation (1), and the MC at each stage of the drying process was determined using Equation (2). After cutting the moisture content specimen, the cross-section of the test piece was scanned with a scanner. The cracking and cross-sectional deformation of the specimen were measured using Image J (1.35k) software. The degree of cross-sectional deformation of the test piece was defined as the difference between the thickness of the cross-section side and the thickness of the most severely concave part [22].

*m*_0_ = *m*/(*W*_o + 1_) (1)

where: *m*_0_ is the oven-dried weight of the entire test piece, g; *m* is the absolute dry weight of the entire test piece, g; *Wo* is the initial MC of the moisture content specimen of the test piece.

*W* = 100 × (*m_i_* − *m*_0_)/*m*_0_ (2)

where: *W* is the MC of the test piece at each stage of the drying process; %; *m_i_* is the weight of the test piece at each stage of the drying process, g; *m*_0_ is the oven-dry weight of the sample, g.

#### 2.3.2. Optimized Drying Process Experimental Methodology

A constant temperature and humidity chamber was used to simulate the drying process of kiln. The wood was sawn into test specimens with dimensions of 50 mm (T) × 25 mm (R) × 350 mm (L). Each group contained 8 pieces, with a total of 4 groups. The samples were dried following the optimized drying schedule determined based on 100 °C drying experimental results. The initial MC of the test pieces was 62.67%.

Following the optimized drying schedule based on 100 °C drying experimental results, test specimens were loaded into the drying kiln after the internal temperature and humidity stabilized at preset levels. The kiln ambient conditions were continuously monitored during the experiment. Specimens were regularly removed and instantly weighed to obtain their real-time MC, and the kiln operating parameters were dynamically adjusted accordingly. During the drying process, the MC of the test pieces was monitored in real time. The moisture distribution and residual stress indicators of the test pieces were measured at MC of 50%, 40%, 30%, 20%, and 10%, respectively. The testing methods were referenced to GB/T 6491-2012 [23] The Drying Quality of Sawn Timber.

#### 2.3.3. Determination of Moisture Content Distribution and Deviation

**Figure 1:**
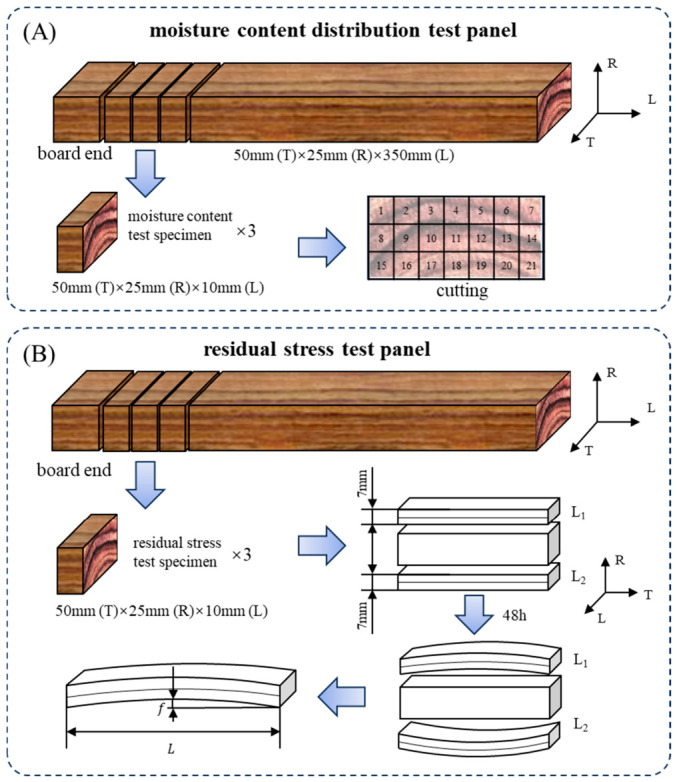
Schematic diagram of sample preparation: (**A**). Schematic diagrams for moisture content test specimen; (**B**). Schematic diagrams for residual stress test specimen.

When the MC of the test piece reached 50%, 40%, 30%, 20%, 10%, the test specimens was took out from the drying equipment. The MC distribution test specimen was cut in accordance with Figure 1A, weighed and recorded, then placed in the oven for oven-drying. After oven-drying, it was weighed again. The MC of each test block was calculated using Equation (2), and the average MC of the specified region of the specimen was determined using Equation (3).

(3) $$\bar{W}=\frac{\sum_{i}^{n}m_{1i}-\sum_{i}^{n}m_{0i}}{\sum_{i}^{n}m_{0i}}$$

where: $\bar{W}$ is immediate average MC of the target region in the moisture distribution test piece; $m_{1i}\;$ is immediate weight of the i-th moisture content test piece in the target region, g; $m_{0i}$ is oven-dried weight of the i-th moisture content test piece in the target region, g; *n* is upper limit serial number of the test pieces in the target region.

The MC deviation in thickness is calculated by Equation (3) to obtain the average MC of the core and the surface, and then the MC deviation of the core-surface layer is derived by Equation (4).

(4) $$\Delta W_{k}=W_{C}-W_{S}$$

where: $\Delta W_{k}$ is the MC deviation of the MC distribution test piece per unit thickness, %; $\;W_{c}$ is the average MC of the core layer of the MC distribution test piece, %; $W_{s}$ is the average MC of the upper and lower surface layers of the layered MC distribution test piece, %.

#### 2.3.4. Determination of Residual Stress

The residual stress indicators of the experimental test pieces was determined by the slice method. When the MC of the test pieces reached the specified values (50%, 40%, 30%, 20%, 10%), the specimens were removed from the drying equipment, as shown in Figure 1B, and then the specimens were cut using a circular saw. The length of the layered test slices was measured using an electronic scanner and Image J software, and the result was recorded as *L*. The stress test specimens were then sealed for 24 h and placed in a well-ventilated area at room temperature for another 24 h. The deflection caused by the deformation of the test slices was measured using the same electronic scanner and Image J software, with an accuracy of 0.001 mm. The residual stress indicator of the slice method (*Y*) was calculated according to Equation (5).

(5) Y=f∕L×100%

where: *Y* is the residual stress indicator, %; *f* is the deflection of the test piece after deformation, mm; *L* is the length of the test specimen, mm.

#### 2.3.5. Determination of Drying Rate

The drying rate of the specimens was calculated using Equation (6) based on the starting and ending MC and the time elapsed between these two points.

(6) $$v=100\times (w_{0}-w_{1})/t$$

where: v is drying rate, %/h; $\;w_{0}$ is the starting MC, %; $w_{1}\;$ is the ending MC, %;  t is drying time, h.

#### 2.3.6. Determination of Warp and Drying Check

Warp includes bow, crook, and cup, all of which are measured as the ratio of the maximum bending height to the horizontal length of the inner curved surface, calculated in accordance with Equation (7).

(7) WP=h/l×100%

where: WP is the warp, %; h is the maximum bending height, mm; l is the length of the inner curved surface, mm.

The longitudinal crack degree of the test piece is defined as the ratio of the total length of longitudinal cracks on its surface to the length of the test piece. This ratio is calculated using Equation (8).

(8) LS=l/L×100%

where: LS is the longitudinal surface check degree, %;  l is the length of longitudinal cracks, mm; L is the length of the test material.

#### 2.3.7. Determination of Color

Different test pieces under various drying schedules were selected as color detection panels, with two pieces in each group. Ten points were selected from each panel. The surface color of specimens was measured with a colorimeter before and after drying in accordance with the coordinate parameters of the CIE-Lab color space system. The total color difference of the test pieces was calculated according to Equations (9)–(12) and the final saturation of the test pieces was calculated according to Equation (13).

(9) $$\Delta L^{*}=L_{1}^{*}-L_{0}^{*}$$

(10) $$\Delta a^{*}=a_{1}^{*}-a_{0}^{*}$$

(11) $$\Delta b^{*}=b_{1}^{*}-b_{0}^{*}$$

(12) $$∆E^{*}=\sqrt{{\left(∆L^{*}\right)}^{2}+{\left(∆a^{*}\right)}^{2}{+\left(∆b^{*}\right)}^{2}}$$

(13) $$C=\sqrt{{\left(a_{1}^{*}\right)}^{2}{+\left(b_{1}^{*}\right)}^{2}}$$

where: $\Delta L^{*}$ is the lightness difference; $\Delta a^{*}$ is the red-green colorimetric difference; $\Delta b^{*}$ is the yellow–blue colorimetric difference; $∆E^{*}$ is the total colorimetric difference, NBS; 1 indicates the value after drying completion; 0 indicates the initial value before the start of drying; C is the saturation and vividness of color.

#### 2.3.8. Statistical Analysis

A one-way analysis of variance (ANOVA) was conducted using IBM SPSS Statistics 27 to assess the significance of differences among groups. When the ANOVA indicated a significant overall effect, Duncan’s multiple range test was employed as a post-hoc analysis to compare the means between specific groups. Statistical significance was defined at *p* < 0.05.

## 3. Results and Discussion

### 3.1. Analysis of the 100 °C Experimental

Based on the experimental results and Table 1, the drying defect grades of *Dalbergia odorifera* are determined as follows: initial cracking at Grade 3, internal cracking at Grade 1, cross-section distortion at Grade 2, twist at Grade 2, and drying rate at Grade 3, as shown in Table 2. The lowest grade was used as the comprehensive grade for the drying defect of *Dalbergia odorifera*, which was designated as Grade 3.

#### 3.1.1. Initial Check and Internal Check

**Figure 2:**
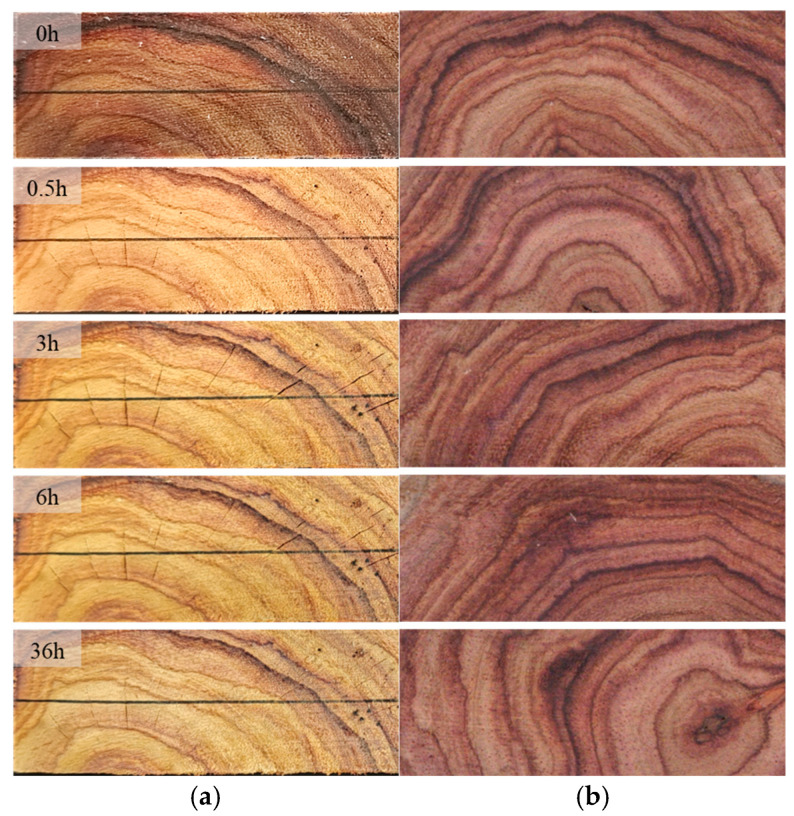
Cracking conditions of test specimens during the drying experiment: (**a**) End checks of lumber at different time intervals; (**b**) Internal checks.

The initial MC of the test piece is 72.61%. During the initial drying stage, the surface of the test piece rapidly loses moisture, leading to a significant moisture gradient between the surface and the interior of the wood. This moisture gradient induces the initial cracking. As shown in Figure 2a, after drying for half one hours, 25% of the test pieces began to exhibit small cracks; after drying for one hour, all test pieces developed small cracks; after drying for two hours, all test pieces showed small end cracks, with only two end surfaces showing the end surface cracks and one end surface showing the splitting crack. The initial cracking reached its maximum extent, and the width of the cracks was all less than 2 mm. At this point, the average MC of the test pieces was 51.43%. After drying for 4 to 5 h, the MC decreased from 39.99% to 35.72%. As internal shrinkage occurred in the wood, the internal stresses were generated, which gradually healed the initial cracks. After drying for 24 to 36 h, the fine end face checks and end face surface checks had been largely healed. The results indicated that the initial cracking grade of *Dalbergia odorifera* ranged from Grade 2 to Grade 3. Among them, the Grade 3 accounted for 5.56%. According to the classification standard for the Baidu experimental method, the initial cracking grade was determined as Grade 3. The initial cracking of the wood or bamboo was primarily influenced by the temperature difference between dry and wet bulbs during the initial drying stage. This difference resulted in MC gradients, leading to drying stress between the material’s surface and its internal layers. [24,25]. The results demonstrated that initial cracking was the primary defect during the drying process of *Dalbergia odorifera* heartwood. When making the drying schedules of *Dalbergia odorifera*, special attention should be paid to maintaining a small temperature difference between dry and wet bulbs in the initial drying stage and controlling the initial temperature to rise slowly.

In the initial stage of drying, longer surface cracks may extend into the interior of the wood to form internal cracks [26]. Primarily occurring in the later stage of drying. It was closely associated with the temperature and humidity conditions in the early stage of drying and the drying temperature at the end of the drying period, while exhibiting minimal correlation with the final drying humidity. After the experiment, no internal cracks were observed in all of the *Dalbergia odorifera* test pieces, as shown in Figure 2b, indicating that the internal crack grade was determined to be Grade 1.

#### 3.1.2. The Deformation of Cross-Section and Twist

The cross-section deformation of *Dalbergia odorifera* heartwood ranged from 0.098 to 0.667 mm, with a drying property grade of 1 to 2. Among these, the Grade 1 accounted for 66.7% and the Grade 2 accounted for 33.3%. The comprehensive grade for cross-section deformation is determined to be Grade 2 (see Table 3). Rapid moisture loss during wood drying generates capillary tension and drying stress, leading to the wood exhibited depression or wrinkling. *Dalbergia odorifera* has a relatively high density and fine texture among broadleaf woods. During the drying process, moisture migration is hindered by the abundant tyloses inside the wood, preventing the excessive moisture loss and thus minimizing the cross-section deformation.

The uneven material density or differential shrinkage in various sections of wood can lead to the deformation after drying. The average warpage of all test panels was 1.93 mm, with a minimum of 0.17 mm and a maximum of 3.92 mm, so the warpage was determined to be Grade 3. The average warpage of tangential cut panels was 1.32 mm, while that of radial cut panels was 3.15 mm. Differences in annual ring morphology and moisture distribution induce distinct drying stress gradients, thereby generating a prominent sectional deformation discrepancy between tangential and center panels. The warpage of tangential cut panels is significantly less than that of radial cut panels and center panels. The final treatments can be applied in the final stage of drying, or the heavy objects can be used to press and reduce distortion and deformation

#### 3.1.3. Drying Rate

**Figure 3:**
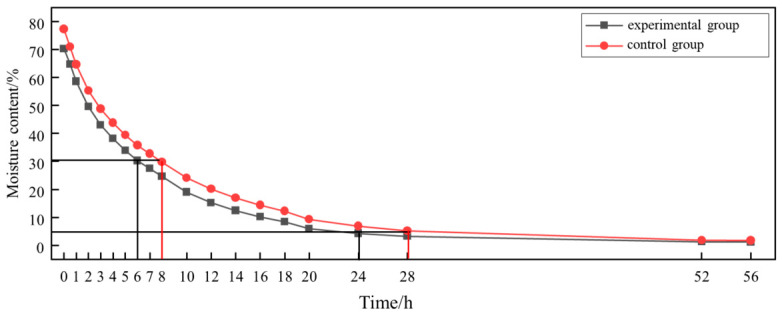
Drying rate of *Dalbergia odorifera* under 100 °C experiment method.

The MC variation of the test specimens in the drying process was shown in Figure 3. The initial average MC of all test specimens was 72.61%, with a total drying duration of 56 h and an average drying rate of 1.27%·h^−1^. For the experimental group, the MC decreased from 70.27% to 30% in 6 h, corresponding to a drying rate of 6.71%·h^−1^; and from 30% to 5% in approximately 18 h, with a drying rate of 1.38%·h^−1^; and from 5% to 1.15% in 32 h, with a drying rate of 0.12%·h^−1^. For the control group, the MC decreased from 77.27% to 30% within 8 h, corresponding to a drying rate of 5.91%·h^−1^; and from 30% to 5% in approximately 20 h, with a drying rate of 1.25%·h^−1^; and from 5% to 1.74% in 28 h, with a drying rate of 0.12%·h^−1^. The overall drying rates of the experimental and control groups were 1.23%·h^−1^ and 1.35%·h^−1^, respectively, which was determined as Grade 3. In the control group, the MC of the tangential cut panels and center panels was relatively higher, but there was no significant difference in drying results between them and the radial cut panels. It is unnecessary to distinguish panel types during kiln drying, and they can be dried under the same conditions.

#### 3.1.4. Drying Schedules Derived from 100 °C Experiment

According to the experimental results, the initial MC of the test pieces was 70.27%. After dring for 0.5 h, a fawned cracks occurred on end faces, followed by extensive cracking was observed after 1 h, and the cracks tended to stabilize after 6 h. Corresponding to the MC at each stage, the cracking was concentrated in the MC ranged from 64.75% to 58.56%, and it reached stability when the MC decreased to approximately 30%. Therefore, the drying parameters were specified as follows: when the MC was above 60%, the dry-bulb temperature was maintained at 60 °C with a wet-bulb depression of 3 °C; when the MC was between 60% and 30%, the temperature should be raised gradually at each stage, and the heating rate can be appropriately increased in subsequent stages to enhance drying efficiency. Based on the table “Relationship between moisture content and wet-dry bulb temperature of broad-leaved wood” in Reference [20], the drying schedule for 25 mm-thick *Dalbergia odorifera* wood was preliminarily established, as shown in Table 4.

#### 3.1.5. Optimized Experimental Drying Schedules Derived from 100 °C Experiment

Wu et al. [18] established the drying schedules for the sapwood of *Dalbergia odorifera* via the Baidu experimental method. The experimental results were largely consistent with those obtained from the heartwood in the present study, which indicated that there was no significant difference in the drying characteristics between the heartwood and sapwood of *Dalbergia odorifera*. Therefore, the results from the 100 °C experimental method were adopted as Schedules 1 for the optimization of drying schedule. The schedules 2 was derived by moderately reducing the initial temperature and final hardness of Schedules 1. The drying schedules formulated by Dong et al. [27] and Liu et al. [10] for precious tropical hardwood species provided crucial references for the conventional drying of *Dalbergia odorifera*. Based on their experimental results, the drying schedules 3 and schedules 4 were optimized and showed in Table 5.

### 3.2. Optimization of Drying Schedules of Dalbergia odorifera

#### 3.2.1. Drying Rate Under Different Drying Schedules

**Figure 4:**
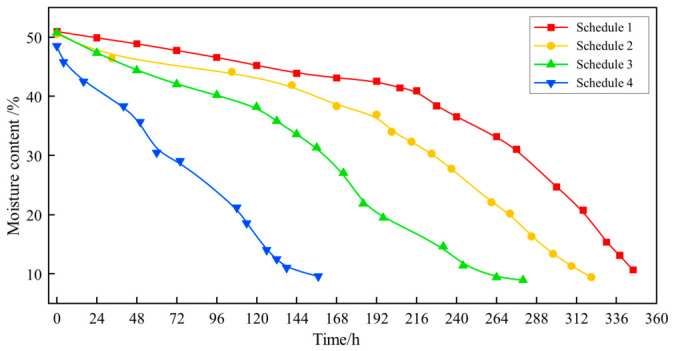
Drying rates of materials under different drying benchmarks.

The MC changes of the *Dalbergia odorifera* specimens under different drying schedules were showed in Figure 4, and the corresponding drying rates of the samples at different stages were presented in Table 6. In the early drying stage (MC 50–40%), the drying rates were 0.05%·h^−1^, 0.06%·h^−1^, 0.11%·h^−1^, and 0.56%·h^−1^, respectively. During the intermediate drying stage (MC 40–20%), the drying rates were relatively consistent, with values of 0.20%·h^−1^, 0.17%·h^−1^, 0.21%·h^−1^, and 0.20%·h^−1^. In the final drying stage (MC 20–10%), the drying rates were 0.34%·h^−1^, 0.22%·h^−1^, 0.13%·h^−1^, and 0.24%·h^−1^, respectively. The results indicated that higher drying temperatures and lower humidity levels were associated with the accelerated drying rates.

Moreover, there were significant differences in the overall drying rates among the different drying schedules. In the early drying stage, the higher temperature and relative humidity resulted in a slower drying rate. For *Dalbergia odorifera*, a high density wood rich in tyloses, the moisture migration from the wood surface was hindered under high-temperature and high-humidity conditions, leading to a reduced drying rate [28]. Therefore, reducing the humidity in the initial stage could enhance the drying efficiency in the early period. In the final drying stage, the removal of bound water from the wood required higher energy input [29]. A higher temperature and a lower humidity resulted in a faster drying rate, thus increasing the temperature difference between the dry-bulb and wet-bulb temperatures could improve the drying efficiency in the final drying stage.

#### 3.2.2. The Influence of the Different Drying Schedules on the Moisture Content Distribution

**Figure 5:**
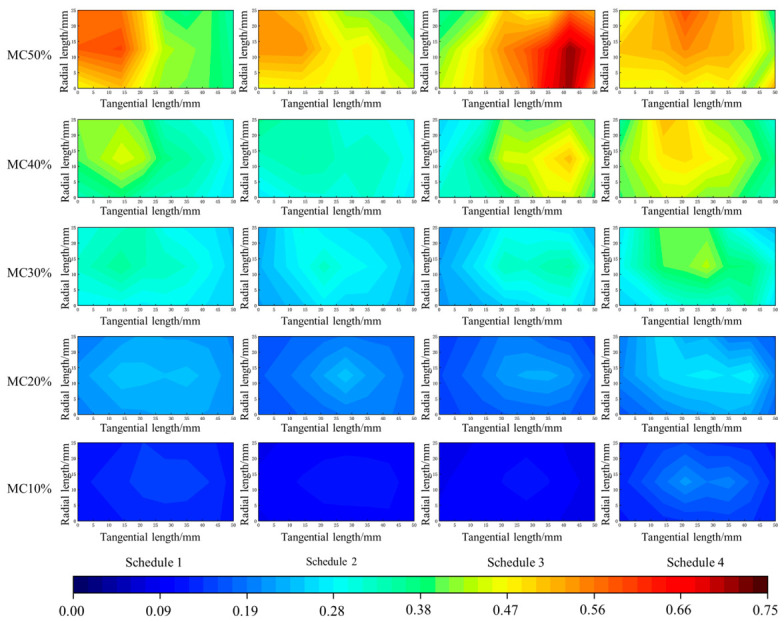
Moisture content distribution of specimens at different drying stages under different drying schedules.

**Figure 6:**
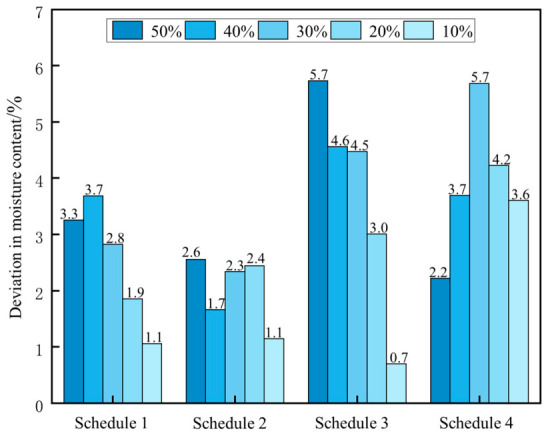
Deviation of moisture content of specimens at different drying stages under different drying schedules.

The MC distribution of test pieces at different MC stages under four drying schedules was presented in Figure 5. The results indicated that when the MC was 50% under different drying schedules, there is an uneven moisture distribution in the test pieces. With the progression of drying, the MC differences among various parts of the test pieces gradually decreased. The MC deviations along the thickness direction of the test pieces at different MC stages under the four drying schedules were illustrated in the Figure 6. It can be observed that when the MC decreased to approximately 20%, the MC within the test pieces tended to be uniform, and the deviation of MC between the heartwood and sapwood layers decreased. Upon completion of drying (when the MC reached 10%), all of the deviation of MC of the test pieces under drying schedules 1, 2, and 3 meet the Grade 1, with values of 1.1%, 1.1%, and 0.7%, respectively. In contrast, the MC deviation of the test pieces under drying schedule 4 was relatively higher, at 3.6%.

When the MC of wood exceeds the fibre saturation point (FSP), most of the water in the wood is lost as free water from the pores and intercellular spaces [30]. The heartwood of *Dalbergia odorifera* was rich in oil and tyloses. The closer to the pith, the denser the cellular structure of the wood. The slow loss of moisture in the middle layer of the specimens leaded to an initially uneven moisture distribution at the early drying stage, which is correlated with the location of heartwood in the specimens [31]. During the middle drying stage, when the MC of wood drops to or below the FSP, the internal temperature gradient and moisture gradient in the test pieces drived the gradual migration of moisture from the central layer to the surface [32]. The differences of MC between the heartwood and sapwood layers gradually decreased, and presented a more uniform moisture distribution. In the final stage of drying, the temperature rised and the humidity decreased, causing the MC of wood to decrease to approximately 9%. At the end of drying schedule 4, the temperature was relatively low and the humidity was high, which led to a weak internal moisture migration capacity of the specimens. The MC differences between the heartwood and sapwood layers remained significant at the end of drying, and the average final MC was relatively higher. Consequently, the dried quality of the specimens was determined to be Grade 4.

#### 3.2.3. Residual Stress Indicators Under Different Drying Schedules

**Figure 7:**
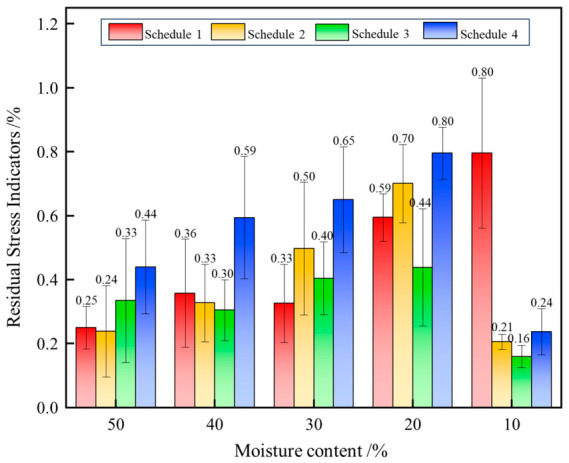
Residual stress of the surface of specimens at different drying stages under different drying schedules.

The variation of residual stress in the upper surface layers of the specimens at different MC stages under four drying schedules was showed in Figure 7. At the initial stage of drying, when the MC of all test pieces reached 50%, the average residual stress indicators for the four schedules were 0.39%, 0.20%, 0.34%, and 0.36%, respectively. Upon completion of drying, with the MC of all test pieces reduced to 10%, the average residual stress were 0.87%, 0.22%, 0.14%, and 0.21%, respectively. Among these, the final residual stress of the drying schedule 3 met the Grade 1 for dried lumber quality. The final residual stress of the schedules 2 and 4 met the Grade 2 for dried lumber quality. In contrast, the final residual stress of drying schedule 1 exhibited the highest due to the relatively higher drying temperature in the final stage of the drying, which did not meet the Grade 2 for dried lumber quality.

During the drying process, the specimens residual stress detection showed that the test pieces underwent tensile plastic deformation. The surface layers were affected by the tensile stress, and the internal regions experienced compressive stress, leading to a relatively higher residual stress. As the drying process proceeded, the specimens underwent compressive plastic deformation: the tensile stress was generated in the internal regions, and the surface layers underwent compressive stress, gradually offsetting the previously formed tensile stress [33], and the residual stress decreased. At the end of the drying process, the residual stress was the net difference between the compressive stress and the previous tensile stress, with the irrecoverable plastic deformation and the residual stress. For all drying schedules, the residual stress of the drying schedules 2, 3, and 4 exhibited the same trend with the decreasing moisture content: they initially increased and then decreased, reaching the minimum values at the MC of 10%. The hardness of specimens for drying schedule 1 was the highest. During the middle and late stages of drying, the MC of specimens decreased rapidly. The compressive stress on the specimens cannot counteract the tensile stress, resulting in a significant irreversible plastic deformation. This leaded to an abnormal residual stress that failed to meet the drying quality standards. Therefore, it is not advisable to use a relatively high temperature during the drying process of *Dalbergia odorifera*, which can avoid the generation of excessive residual stress.

#### 3.2.4. Grading of Drying Quality Under Different Drying Schedule

The drying quality parameters were measured after drying under each drying schedule, and the results were presented in Table 7. According to the standards, the average MC grade, MC deviation grade, residual stress grade, warpage grade, longitudinal crack grade, and internal crack grade of 25 mm-thick of *Dalbergia odorifera* specimens under each drying schedule are summarized in Table 8. A comprehensive evaluation of drying quality indicated that the drying grades of the four drying schedules are 3, 2, 2, and 4, respectively.

#### 3.2.5. The Effect of Different Drying Schedules on the Color of Specimens

**Figure 8:**
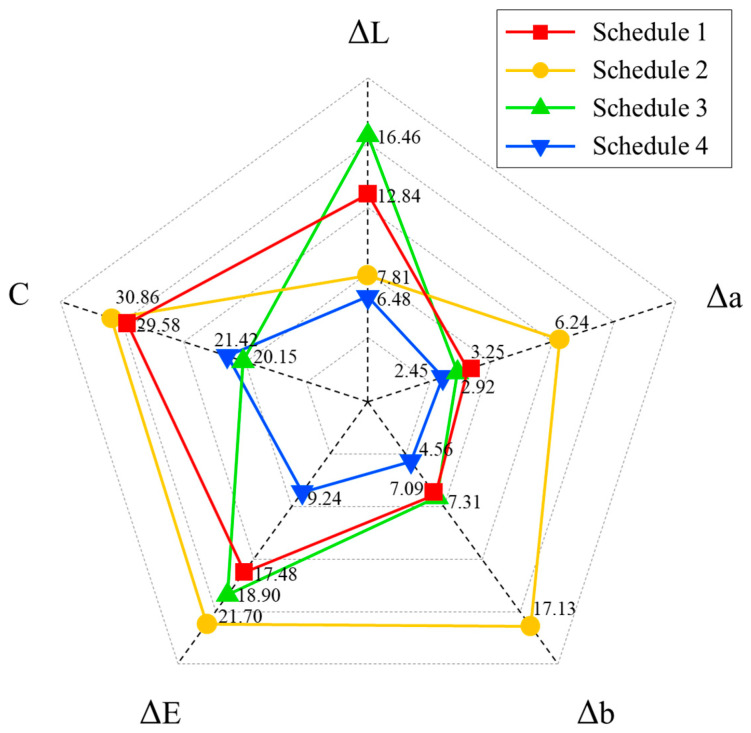
Color variation of lumber under different drying schedules.

The color of wood was a critical indicator for evaluating the value of *Dalbergia odorifera* in the current redwood market. The color comparison of the heartwood of *Dalbergia odorifera* before and after drying under different drying schedules was presented in Table 9. The wood color changed uniformly from purplish-red to reddish-brown, shifting from a darker hue to a lighter one [34]. The wood color uniformly changed from purplish-red to reddish-brown, shifting from the darker shade to the lighter one. The color difference variations of the test pieces before and after drying under each drying schedule, and the comparison of the saturation values of the test pieces after drying were showed in Figure 8.

As shown in Figure 8, after drying for schedule 1, the values of $\Delta a^{*}$ and $\Delta b^{*}$ were relatively low, while the $\Delta L^{*}$ was relatively high, resulting in a $∆E^{*}$ value of 17.48. The specimens dried under schedule 1 exhibited high saturation and desirable color performance. Under drying schedule 2, the values of $\Delta a^{*}$ and $\Delta b^{*}$ showed significant variations, while the $\Delta L^{*}$ remained relatively stable. Notably, the E value under schedule 2 was the highest among all schedules, and the corresponding specimens achieved the greatest color saturation. For drying schedule 3, the values of $\Delta a^{*}$ and $\Delta b^{*}$ of specimens showed minor fluctuations, with $\Delta L^{*}$ exhibiting the most pronounced change, and the $∆E^{*}$ value was 18.90. The specimens dried under this schedule exhibited the lowest color saturation, measured at only 20.15. Under drying schedule 4, all of three parameters ($\Delta a^{*}$, $\Delta b^{*}$ and $\Delta L^{*}$) were relatively small, with a $∆E^{*}$ value of 9.24. The color saturation of the specimens after drying was also low, resulting in a dull appearance. In the current market of rosewood, the *Dalbergia odorifera* with richer, more reddish and more purplish hues was considered more popular and had a higher value. The specimens dried under drying schedule 2 exhibited a slightly brighter color, with the highest degree of redness and yellowness, thus achieving the best color effect after drying.

## 4. Conclusions

In this study, the 100 °C experimental method was adopted to determine the drying schedules for *Dalbergia odorifera*. On this basis, the different drying schedules were designed to conduct the optimization experiments.

The 100 °C experiment indicated that *Dalbergia odorifera* achieved an overall Grade-3 drying grade, with Grade-3 initial cracking and drying rate (1.38%·h^−1^), as well as Grade-2 cross-sectional and torsional deformation.

Through comparing different drying schedules, the optimal drying parameters for 25-mm-thick 17-year-old plantation-grown *Dalbergia odorifera* were determined: initial 48 °C with 3 °C wet-bulb depression and final 68 °C with 12 °C wet-bulb depression, which requires around 12 days to reduce moisture content from 50% to 10%. These results provide theoretical references for its industrial drying.

This optimized schedule satisfies standard drying quality by balancing efficiency and color stability. Future studies are suggested to explore timber with different dimensions and stand ages, develop composite drying technologies combined with heat treatment, and reveal synergistic mechanisms, so as to perfect drying theories and promote industrial application of plantation-grown *Dalbergia odorifera*.

## Figures and Tables

**Table 1:** Classification of drying defect and drying rate in 100 °C experimental.

Grade	Initial Check	Internal Check	Cross-Section Distortion/mm	Twist/mm	Drying Rate/h
1	No cracks or only end check	No	≤0.5	≤0.5	≤10
2	Short-end checks and surface check	Fine checks (≤4) or one wide check	0.6~1.0	0.6~3.0	11~15
3	Long-end checks or splits or short-end fine surface checks (≤10)	Fine cracks (2~4) or fine check (5~9) or wide check (1~2) combined with fine check (5~9)	1.1~2.0	3.1~6.0	16~20
4	Short-end fine surface check (≤10), long-end fine surface checks or wide surface checks (≥5)	wide checks (5–8), fine checks (10–15), or wide checks (2–4) combined with fine checks (5–9)	2.1~3.5	6.1~9.0	21~30
5	Long-end fine surface cracks or wide surface checks (≥5)	wide check (≥8) or fine checks (≥15) or wide checks (5–8) combined with fine checks (10–15)	≥3.6	≥9.1	≥30

Note: 1. Drying Rate Classifications are determined based on the time required for the MC of wood to decrease from 30% to 5%; 2. Surface checks are classified as short if their length is ≤5 cm, and long if their length is ≥5 cm; they are classified as fine if their width is ≤2 mm, and wide if their width is >2 mm. The same classification criteria apply to internal checks.

**Table 2:** Evaluation of drying defect level of *Dalbergia odorifera*.

Check	Initial Check	Internal Check	Cross-Section Distortion	Twist Grade	Drying Rate	Comprehensive Drying Defect Grade
grade	3	1	2	3	3	3

**Table 3:** The deformation values of the cross section and twisting of *Dalbergia odorifera*.

Specimen No.	Cross-Section Deformation/%	Mean Value	Torsion Value/mm	Mean Value
1	0.098	0.352	1.57	1.32
2	0.129		0.17	
3	0.286		2.23	
4	0.667		1.33	
5	0.348		2.20	
6	0.581		0.49	
7	0.375	0.508	2.07	3.15
8	0.648		3.48	
9	0.500		3.92	

**Table 4:** Drying schedules for *Dalbergia odorifera* under 100 °C experiment method.

Stage	Moisture Content/%	Dry-Bulb Temperature/°C	Wet-Bulb Depression/°C	Relative Humidity/%	Equilibrium Moisture Content (EMC)/%
1	≥50	60	3	90	16
2	50~40	62	3	88.5	16
3	40~35	64	4	83	14
4	35~30	68	6	78	12
5	30~25	75	8	70	9.5
6	25~20	80	10	66	8.5
7	20~15	85	18	51	6
8	≤15	90	25	32	3.5

**Table 5:** Optimization of the Drying Schedules Employed in the Experiment.

Drying Schedules	Initial Temperature/°C	Initial Wet-Dry Bulb Depression/°C	Final Temperature/°C	Final Wet-Dry Bulb Depression/°C
1	60	3	80	15
2	54	3	74	12
3	48	3	68	12
4	40	2	60	12

**Table 6:** Moisture content and drying rates of specimens under different drying schedules.

Schedule	Initial MC /%	Final MC /%	Drying Rate (%/h)			
			MC50–40%	MC40–20%	MC20–10%	Overall (MC50–10%)
1	63.33	6.15	0.05	0.20	0.34	0.12
2	62.53	9.43	0.06	0.17	0.22	0.13
3	67.52	8.93	0.11	0.21	0.13	0.15
4	57.32	9.53	0.56	0.20	0.24	0.25

**Table 7:** Drying quality parameters under different drying schedules.

Drying Schedules	Average MC%	Deviation of MC in Thickness/%	Residual Stress Indicator/% (10%)	Warp Degree/%	Longitudinal Surface Check Degree/%	Internal Check
1	6.15	1.06	0.87	0.26	0.00	0
2	9.43	1.15	0.22	0.41	0.00	0
3	8.93	0.70	0.14	0.09	0.00	0
4	9.53	3.60	0.21	0.27	0.80	0

**Table 8:** The drying quality grade evaluation of specimens.

Grading of Drying Quality	Average MC%	Deviation of MC in Thickness/%	Residual Stress Indicator/% (Slicing)	Warp Degree/%	Longitudinal Surface Check Degree/%	Internal Check
Grade 1	6~8	2.0	≤0.16	2.0	4	inadmissibility
Grade 2	8~12	2.5	≤0.22	4.0	6	inadmissibility
Grade 3	12~15	3.0	No inspection required	6.0	10	inadmissibility
Grade 4	20	No inspection required	No inspection required	2.0	4	inadmissibility

**Table 9:** The comparison of color of specimens before and after drying under different drying schedules.

Time	Schedules 1	Schedules 2	Schedules 3	Schedules 4
Before drying	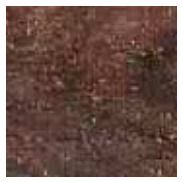	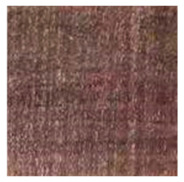	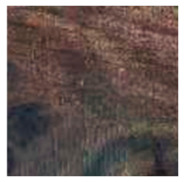	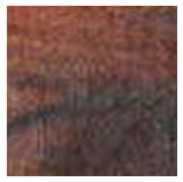
After drying	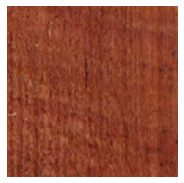	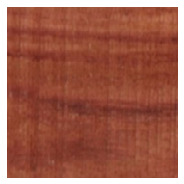	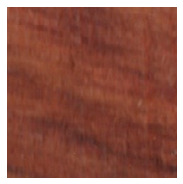	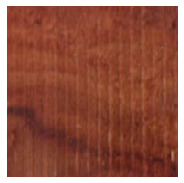

## Data Availability

The original contributions presented in this study are included in the article. Further inquiries can be directed to the corresponding authors.
